# Quantification of the Ability of Natural Products to Prevent Herpes Virus Infection

**DOI:** 10.3390/medicines7100064

**Published:** 2020-10-06

**Authors:** Kunihiko Fukuchi, Hiroshi Sakagami, Yoshiaki Sugita, Koichi Takao, Daisuke Asai, Shigemi Terakubo, Hiromu Takemura, Hirokazu Ohno, Misaki Horiuchi, Madoka Suguro, Tomohiro Fujisawa, Kazuki Toeda, Hiroshi Oizumi, Toshikazu Yasui, Takaaki Oizumi

**Affiliations:** 1 Graduate School of Health Sciences, Showa University, Hatanodai 1-5-8, Shinagawa, Tokyo 142-8555, Japan; kfukuchi@med.showa-u.ac.jp; 2 Research Institute of Odontology (M-RIO), Meikai University, Keyakidai 1-1, Sakado, Saitama 350-0283, Japan; 3 Department of Pharmaceutical Sciences, Faculty of Pharmacy and Pharmaceutical Sciences, Josai University, Keyakidai 1-1, Sakado, Saitama 350-0295, Japan; sugita@josai.ac.jp (Y.S.); ktakao@josai.ac.jp (K.T.); 4 Department of Microbiology, St. Marianna University School of Medicine, Sugao 2-16-1, Miyamae, Kawasaki 216-8511, Japan; asai@marianna-u.ac.jp (D.A.); biseibutsu-001@marianna-u.ac.jp (S.T.); takeh@marianna-u.ac.jp (H.T.); 5 Maruzen Pharmaceuticals Co., Ltd., Fukuyama, Hiroshima 729-3103, Japan; h-ohno@maruzenpcy.co.jp; 6 Daiwa Biological Research Institute Co., Ltd., Sakado 3-2-1, Takatsu-ku, Kawasaki, Kanagawa 213-0012, Japan; m_horiuchi@daiwaseibutsu.co.jp (M.H.); m_suguro@daiwaseibutsu.co.jp (M.S.); t_fujisawa@daiwaseibutsu.co.jp (T.F.); k_toeda@daiwaseibutsu.co.jp (K.T.); h_oizumi@daiwaseibutsu.co.jp (H.O.); takaakio@daiwaseibutsu.co.jp (T.O.); 7 Division of Oral Health, School of Dentistry, Meikai University, Keyakidai 1-1, Sakado, Saitama 350-0283, Japan; yasui@dent.meikai.ac.jp

**Keywords:** Kampo formulae, alkaline extract of *Sasa* sp., pine cone extract, povidone-iodine, HSV, HIV, loss of infectivity, solubilization method

## Abstract

**Background:** Herpes simplex virus (HSV) is usually dormant and becomes apparent when body conditions decline. We investigated the anti-HSV activity of various natural and synthetic compounds for future clinical application. **Methods:** Mock- and HSV-infected Vero cells were treated for three days with various concentrations of samples. For short exposure, 100-fold concentrated virus were preincubated for 3 min with samples, diluted to normal multiplicity of infection (MOI), before the addition to the cells. Anti-HSV activity was evaluated by the chemotherapy index. **Results:** Alkaline extracts of the leaves of *Sasa* sp. (SE) and pine cone (PCE) showed higher anti-HSV activity than 20 Japanese traditional herb medicines (Kampo formulas), four popular polyphenols, and 119 chromone-related compounds. Exposure of HSV to SE or PCE for 3 min almost completely eliminated the infectivity of HSV, whereas much longer exposure time was required for Kakkonto, the most active Kampo formulae. Anti-HSV activity of PCE and Kakkonto could be detected only when they were dissolved by alkaline solution (pH 8.0), but not by neutral buffer (pH 7.4). Anti-HSV activity of SE and povidone iodine was stable if they were diluted with neutral buffer. **Conclusions:** The present study suggests the applicability of SE and PCE for treatment of oral HSV and possibly other viruses.

## 1. Introduction

In the oral cavity, there are many viruses including norovirus, rabies, human papillomavirus, Epstein–Barr virus, herpes simplex viruses (HSVs), hepatitis C virus, and human immunodeficiency virus (HIV). Viral infections have been diagnosed using an oral sample (e.g., saliva mucosal transudate or an oral swab) based on the correlation of HIV anti-IgG/sIgA detection with saliva and serum samples [1]. Oral herpes viruses, HSV-1 and HSV-2, are very common and infectious, and debilitate patients, affect oral health, and have important psychological implications. The therapies currently used for the treatment of HSV infection are pharmacological, topical, systemic, or instrumental, occasionally with laser devices [2]. Many natural products have been investigated for their anti-HSV activity in vitro or in vivo. These include low molecular weight polyphenols [3,4,5,6], water-extracts [7,8,9,10] including Japanese traditional herb medicine (Kampo formulae) [11,12], and alkaline extracts [13] including a lignin-carbohydrate complex [14,15,16,17].

We have already reported the anti-HSV activity of five plant extracts, 13 tannin-related compounds determined by plaque assay [18], and the anti-HSV activity of eight licorice root extracts, 10 licorice flavonoids (including isoliquiritin apioside), five polymethoxyflavonoids (including tricin), and five polyphenols (including epigallocatechin gallate, chlorogenic acid, *p*-coumaric acid, curcumin, and resveratrol) determined by the 3-(4,5-dimethylthiazol-2-yl)-2,5-diphenyltetrazolium bromide (MTT) method [19]. Quantitative structure-activity relationship (QSAR) analysis of these 19 polyphenols and 1705 chemical descriptors demonstrated that their anti-HSV activity correlated well with six chemical descriptors that represent polarizability (MATS5p, GATS5p), ionization potential (GATS5i), number of ring systems (NRS), atomic number (J_Dz(Z)) and mass (J_Dz(m) (r^2^ = 0.684, 0.627, 0.624, 0.621, 0.619, and 0.618, respectively, *p* < 0.0001) [19]. However, most of lower molecular weight polyphenols showed very low anti-HSV-activity.

In the present study, we report the in vitro anti-HSV activity of 20 Kampo formulas and alkaline extracts of the leaves of *Sasa* sp. (SE) and pine cone combined with dextrin (PCE), representative polyphenols, and a total of 119 chromones, esters, and amides [20,21,22,23,24,25,26], synthesized from chromone (to search for new type of anti-HSV agents), a back-bone structure of flavonoids, together with positive control acyclovir [27], representative polyphenols (resveratrol, *p*-coumaric acid, and curcumin used as negative controls) [19] and povidone iodine (PVP-I), a popular gargle [28].

Since gargling time with mouth wash is usually a minute order, we investigated whether short exposure of HSV (1.5 or 3 min) is enough to inactivate HSV. Since PCE and the Kampo formula contain many acidic substances such as a lignin-carbohydrate complex and its degradation products, it is expected that an alkaline solution may be useful to extract the active substances in higher yield compared with the neutral buffer, although some elevation of degradation would be inevitable. Therefore, we also compared the anti-HSV activity and its stability using either an alkaline solution (1.39% NaHCO_3_, pH 8.0) or a neutral buffer [phosphate-buffered saline (PBS), pH 7.4].

## 2. Materials and Methods 

### 2.1. Materials

The following chemicals and reagents were obtained from the indicated companies: Eagles minimum essential medium (MEM) (Gibco BRL, Grand Island, NY, USA); fetal bovine serum (FBS), 3-(4,5-dimethylthiazol-2-yl)-2,5-diphenyltetrazolium bromide (MTT), resveratrol, azidothymidine (AZT), 2’,3’-dideoxycytidine (ddC) (Sigma-Aldrich Inc., St. Louis, MO, USA); dimethyl sulfoxide, dextran sulfate (DS) (5 kDa) (Wako Pure Chemical Ind., Ltd., Osaka, Japan); acyclovir, curcumin, trans *p*-coumaric acid (Tokyo Chemical Industry Co. Ltd., Tokyo, Japan); tricin (Carbosynth Ltd., Berkshire, UK); curdlan sulfate (79 kDa) (Ajinomoto Co., Inc., Tokyo, Japan); and PVP-I (Showa Seiyaku Co. Ltd., Tokyo, Japan). Twenty Kampo formula (Table 1) were provided by Tsumura & Co, Tokyo, Japan. Culture plastic dishes and plates (96-well) were purchased from Becton Dickinson Labware (Franklin Lakes, NJ, USA).

### 2.2. Preparation of Sasa sp. (SE)

**Figure 1:**
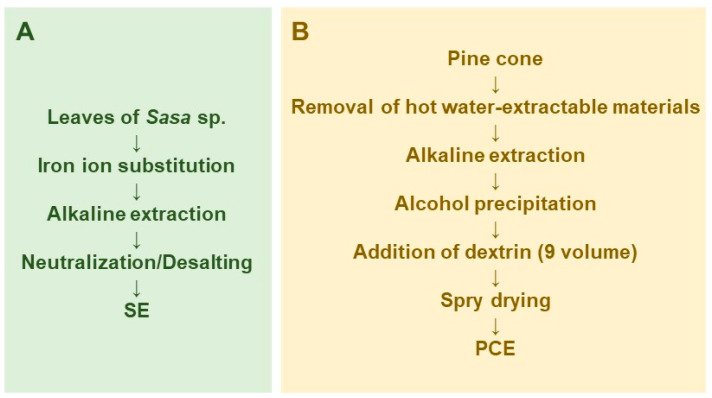
Scheme for large scale preparation of *Sasa* sp. (SE) (**A**) and pine cone extract (PCE) (**B**).

SE was prepared by iron ion substitution, alkaline extraction, and neutralization/desalting (Figure 1A). Lyophilization and measurement of the dry weight of SE showed that it contained 58.2 ± 0.96 mg solid materials/mL [29]. The components of SE are shown in our previous review article [30].

### 2.3. Preparation of Pine Cone Extract (PCE)

Pine cone extract was prepared by modification of the original method of preparation of the lignin-carbohydrate complex [31,32]. In brief, pine cone of *Pinus parviflora* Sieb et Zucc. was washed by hot water extract to remove contaminants and hot-water extractable materials, and then extracted with 0.15 N NaOH to obtain the lignin-carbohydrate complex. The lignin-carbohydrate complex was recovered by ethanol precipitation, and separated from salts and fat-soluble degradation products such as phenylpropanoids. Nine volumes of dextrin were added and spry dried to yield PCE (Figure 1B).

### 2.4. Preparation of Chromones, Esters, and Amides

Twenty four 2-azolylchromone derivatives (E) were synthesized by the conjugated addition reaction of 3-iodochromone derivatives with various azoles [20]. Seventeen 3-benzylidenechromanone derivatives were synthesized by base-catalyzed condensation of the corresponding 4-chromanone with substituted benzaldehyde derivatives [21]. Fifteen chalcone derivatives were synthesized by base-catalyzed condensation of the corresponding acetophenones with various benzaldehyde derivatives [22]. Ten cinnamic acid phenethyl esters were synthesized by the condensation of cinnamic acid and its analogs such as caffeic acid, ferulic acid, and *p*-coumaric acid with the corresponding phenethyl alcohols [23]. Ten 3-flavene derivatives were synthesized by the reductive intramolecular cycloaddition reaction of 2-hydroxychalcone derivatives [22]. Eleven piperic acid amides were synthesized by the condensation of the acid chloride of piperic acid with various amines. Piperic acid was prepared by alkaline hydrolysis of piperine [24]. Eighteen 2-styrylchoromone derivatives were synthesized by base-catalyzed condensation of the corresponding 2-methylchromones with selected benzaldehyde derivatives [25]. Fourteen 3-styrylchoromone derivatives were synthesized by Knoevenagel condensation of the corresponding 3-formylchromones with various phenylacetic acid derivatives [26]. All compounds were dissolved in DMSO at 40 mM and stored at −20 °C before use.

### 2.5. Assay for Anti-Herpes Simplex Virus (HSV) Activity

**Figure 2:**
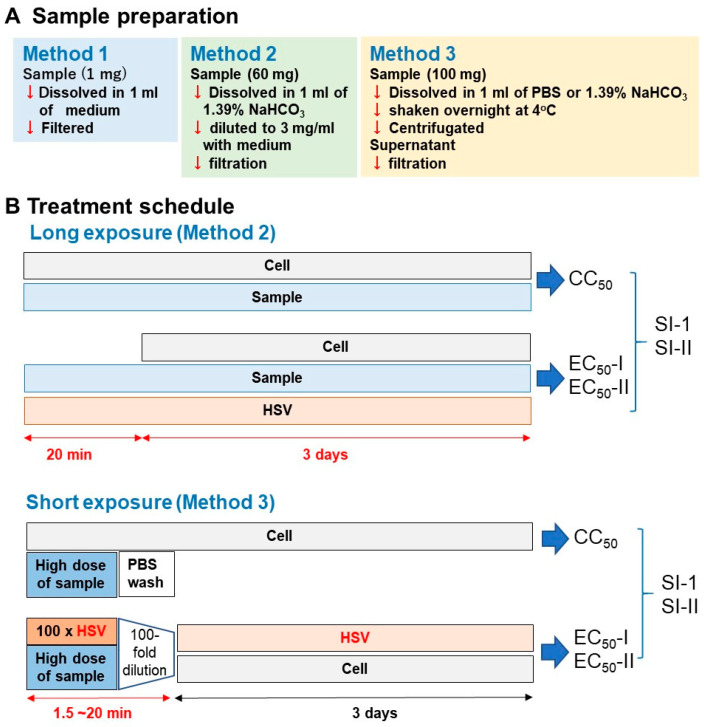
Experimental protocol. (**A**) Long treatment. (**B**) short treatment.

We dissolved the samples using the following three methods. (i) Method 1: Sample was dissolved at 1 mg/mL with culture medium (MEM + 10% FBS) and then sterilized by passing through a Millipore filter (pore size: 0.45 μm); (ii) Method 2: Sample was dissolved at 60 mg/mL with 1.39% NaHCO_3_ (pH 8.0) and then diluted to 3 mg/mL with medium and filtered; (iii) Method 3: Sample was dissolved at 100 mg/mL with phosphate-buffered saline (PBS, pH 7.4) or 1.39% NaHCO_3_, vortexed, and shaken overnight at 4 °C. After centrifugation, the supernatant was collected and then filtered (Figure 2A).

For the long treatment schedule (upper column in Figure 2B), Vero cells, isolated from the kidney of African green monkey (*Cercopithecus aethiops*) were infected with HSV-1 (multiplicity of infection (MOI) = 0.01). HSV-1 and test samples were mixed and stood for 20 min, and the mixture was then added to the adherent Vero cells. After incubation for three days, the relative viable cell number was determined by the MTT reagent.

For the short treatment schedule (lower column in Figure 2B), 100-fold concentrated HSV (MOI = 1) was mixed with samples and stood for 1.5, 3, or 20 min. Then, virus concentration was reduced to 1/100 (MOI = 0.01), added to the cells and incubated for three days. Mock-infected cells were first treated for 1.5, 3, or 20 min with the same concentrations of test samples without HSV, then the sample was removed by suction, washed once with PBS, and incubated for three days in the fresh culture medium. The viability of both HSV-infected and mock-infected cells was determined by the MTT method as described above.

**Figure 3:**
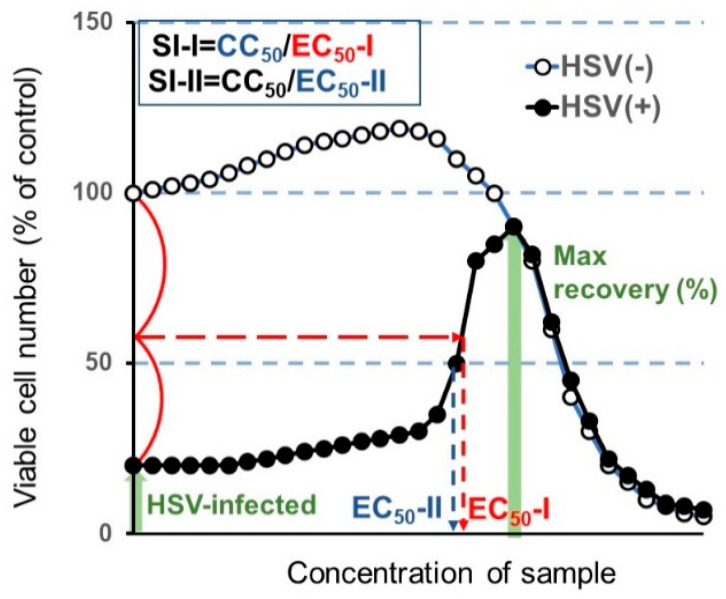
Calculation of anti-herpes simplex virus (HSV) activity.

From the dose-response curve, 50% cytotoxic concentration (CC_50_) in mock-infected cells, and the 50% effective concentration (EC_50_) in HSV-infected cells were determined. EC_50_-I was defined as the concentration at which the viability was restored to the midpoint between that of HSV-infected cells and that of mock-infected cells. EC_50_-II was defined as the concentration at which the viability was restored to 50% of that of the mock-infected cells. The anti-HSV activity was evaluated by the selectivity index (SI-I and SI-II), which was calculated using the following equation: SI-I = CC_50_/EC_50_-I; SI-II = CC_50_/EC_50_-II (Figure 3).

### 2.6. Assay for Anti-Human Immunodeficiency Virus (HIV) Activity

Human T-cell leukemia virus I (HTLV-I)-bearing CD4-positive human T-cell line MT-4, established by Dr. Miyoshi [33], was cultured in RPMI-1640 medium supplemented with 10% FBS and infected with HIV-1_IIIB_ at a multiplicity of infection (MOI) of 0.01. HIV- and mock-infected MT-4 cells (3 × 10^4^ cells/96-microwell) were incubated for five days with different concentrations of extracts and the relative viable cell number was determined by the MTT assay. The concentration that reduced the viable cell number of the uninfected cells by 50% (CC_50_) and the concentration that increased the viable cell number of the HIV-infected cells to 50% of the control (mock-infected, untreated) cells (EC_50_) was determined from the dose-response curve with mock-infected and HIV-infected cells, respectively. The anti-HIV activity was evaluated by the selectivity index (SI), which was calculated using the following equation: SI = CC_50_/EC_50_ [34,35]. Since the viable cell number of HIV-treated cell reached the baseline (zero), the EC_50_-I and SI-I were nearly identical to EC_50_-II and SI-II, respectively.

### 2.7. Statistical Treatment

Experimental values were expressed as the mean ± standard deviation (SD). The statistical significance between the groups was assessed with analysis of variance (ANOVA) followed by Dunette’s multiple comparison test. A *p* value less than 0.05 was considered significant.

## 3. Results

### 3.1. Establishment of Assay Condition for Anti-HSV Activity

**Figure 4:**
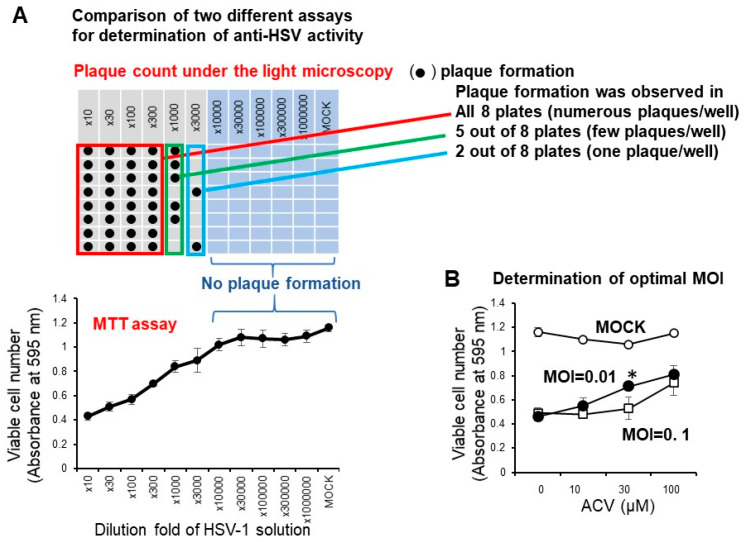
(**A**) Confirmation of correlation of optical density measurement with the MTT method and plaque counting. Vero cells (10,000 cells) were inoculated on a 96-microwell plate and incubated overnight at 37 °C. HSV-1 solution at the indicated dilution fold was then added. After incubation for three days, viable cell number (absorbance at 595 nm) was determined by the MTT method, and plaque formation was counted by light microscopy. (**B**) Effect of different MOI on the anti-HSV activity of ACV. Each value represented as mean ± S.D. was determined (n = 3). Significant difference between MOI = 0.01 and MOI = 0.1 (*p* < 0.05).

We have reported that the anti-HSV activity of most lower molecular weight polyphenols as assessed by SI value (CC_50_/EC_50_) was very low (SI < 1) when compared with the positive control (acyclovir and tricin) (SI > 27.3, 7.1) [19]. We conducted the accurate determination of anti-HSV activity of natural products with low anti-HSV activity using two different plates: 96-well (for CC_50_ determination by the MTT method) and 6-well plates (for EC_50_ determination by plaque assay) may be difficult, especially for short exposure experiments that require quick medium change. Therefore, it was necessary to first investigate whether the infectivity of HSV-1 measured by the MTT method with the 96-well plate correlated with the plaque count of the 6-well plate under the light microscopy. We found that this was the case. With the dilution fold of the virus solution increased, the viable cell number was increased, reaching the plateau level where no plaque formation was observed (Figure 4A). We found that the virus titer of MOI = 0.01 was much better than MOI = 0.1 for the quantitative determination of the anti-HSV of acyclovir (ACV), one of the positive controls used in this study (Figure 4B). When we used MOI = 0.1, accurate evaluation of the viable cell number was difficult due to the presence of too much virus. Therefore, we used the fixed MOI = 0.01 during the cell treatment period to maintain the viability of HSV-infected Vero cells at 20 ~ 40% (Figure 3). The selection of three days was the best incubation time for the determination of anti-HSV activity. We had to use 100-fold concentrated HSV solution (MOI = 1) to treat the virus with extremely higher concentrated samples. We reduced the MOI 100-fold during the days of cell culture (MOI = 0.01).

### 3.2. Anti-HSV Activity of Natural Products

#### 3.2.1. Hot-Water Extract (Kampo Formula) and Alkaline Extracts (SE, PCE)

It was important to first establish the method for dissolving the Kampo formulas. As a first step, we directly mixed 20 Kampo formulas with culture medium (MEM + 10% FBS), and then sterilized them by passing through a Millipore filter (Method 1 in Figure 2A). Both mock-infected and HSV-infected cells were incubated for three days without or with various concentrations of samples, and the viable cell number was determined by the MTT method. From the dose-response curve, we determined the 50% cytotoxic concentration (CC_50_) and 50% protective concentration (EC_50_-I, EC_50_-II) and maximum cell recovery (%) (MCR). The anti-HSV activity was assessed as the SI value (SI-I = CC_50_/EC_50_-I or SI-I = CC_50_/EC_50_-II) (Figure 3). It was unexpected that any Kampo formula, except for S19 (SI-I > 2.2; SI-II > 5.9) did not reduce the cytopathic effect of HSV infection (Supplementary Figure S1). We thought that the failure to detect anti-HSV activity may be due to the interaction of Kampo ingredients and medium components. Based on these data, we did not choose method I to solubilize the Kampo formula. We found that higher anti-HSV activity of Kampo formulas was recovered by dissolving them with 1.39% NaHCO_3_ (pH 8.0) than with PBS (pH 7.4) (Supplementary Table S1, Supplementary Figure S2). Therefore, we dissolved all Kampo formula with 1.39% NaHCO_3_ to make the initial concentration of 60 mg/mL, diluted with culture medium to make a 3 mg/mL solution, and sterilized by passing through a Millipore filter (Method 2 in Figure 2A).

**Figure 5:**
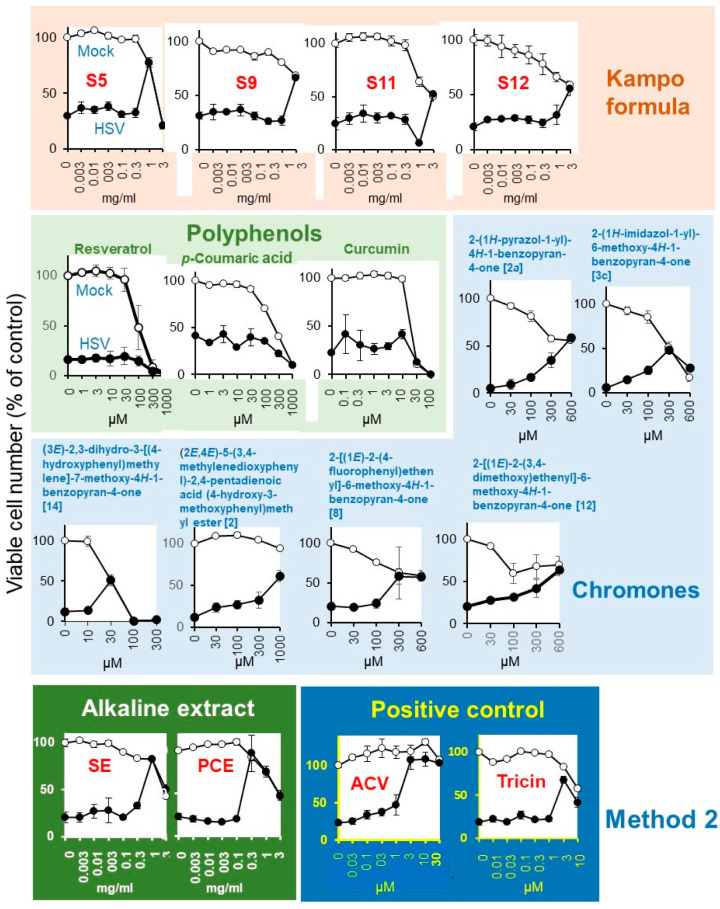
Anti-HSV activity of Kampo formulas, SE, PCE, and acyclovir (ACV). Mock-infected (○) and HSV-infected (●) cells were treated for three days and viable cell number [%t of control (untreated, uninfected cells)] were determined. Each value is represented as mean ± S.D. (n = 3).

Mock-infected and HSV-infected cells were incubated with increasing concentrations of samples and determined for viability (Figure 5). Among the 20 Kampo formulas (S1 ~ S20), Kakkonto (S5) showed the highest anti-HSV activity (SI-1 > 5.2; SI-II > 5.4; MCR = 78%), followed by Yokukansan (S9) (SI-I > 1.0; SI-II > 1.6; MCR = 66%), Yokuininto (S12) (SI-II = 1.3; MCR = 52%) > Jumihaidokuto (S11) (SI-II = 1.1; MCR = 52%). However, their anti-HSV activity was much lower than that of the alkaline extract of *Sasa* sp. (SE) (Supplementary Table S2) (SI-1 = 4.5; SI-II = 6.8; MCR = 90%), alkaline extract of pine cone of *Pinus parviflora* Sieb. et Zuc. (PCE) (SI-1 = 13.1; SI-II = 14.7; MCR = 82%) and acyclovir (ACV) (SI-1 > 23.1; SI-II > 27.3; MCR = 108%) (Table 2).

#### 3.2.2. Polyphenols and Chromone-Related Compounds

Among the four polyphenols, resveratrol (MCR = 20%), *p*-coumaric acid (MCR = 43%), and curcumin (MCR = 42%) showed little or no anti-HSV activity, whereas tricin (SI-II = 7.1; MCR = 68%) showed weak anti-HSV activity (Figure 5, Table 2).

Among the 119 chromone-related compounds, only 2-(1*H*-pyrazol-1-yl)-4*H*-1-benzopyran-4-one (2a), 2-(1*H*-imidazol-1-yl)-6-methoxy-4*H*-1-benzopyran-4-one (3c), (3*E*)-2,3-dihydro-3-[(4-hydroxyphenyl)methylene]-7-methoxy-4*H*-1-benzopyran-4-one (14), (2*E*,4*E*)-5-(3,4-methylenedioxyphenyl)-2,4-pentadienoic acid (4-hydroxy-3-methoxyphenyl)methyl ester (2), 2-[(1*E*)-2-(4-fluorophenyl)ethenyl]-6-methoxy-4*H*-1-benzopyran-4-one (8), and 2-[(1*E*)-2-(3,4-dimethoxy)ethenyl]-6-methoxy-4*H*-1-benzopyran-4-one (12) showed weak anti-HSV activity (Figure 5). Chromone derivatives with higher antitumor activity (assessed with tumor-specificity determined by the ratio of mean CC_50_ against four human oral squamous cell carcinoma cell lines (Ca9-22, HSC-2, HSC-3, HSC-4) to that for three normal oral cells such as human gingival fibroblasts, human periodontal ligament fibroblasts, and pulp cells (indicated by red color) showed no anti-HSV activity. Similarly, chromones with higher anti-HSV activity did not have higher antitumor activity (Supplementary Table S3).

### 3.3. Augmentation of Antiviral Potential of Alkaline Extracts by Reducing the Treatment Time

#### 3.3.1. Rapid HSV Inactivation by SE and PCE 

Since SE and PCE showed approximately 10-fold higher anti-HSV activity (SI-II = 6.8, 14.7) than the twenty Kampo formulas (mean SI-II < 1.1), 6-fold higher than four polyphenols (mean SI-II = 1.9), and 6-fold higher than five of the most potent chromones (mean SI-II = 1.7) (Table 2), we next investigated whether short exposure of HSV to these samples could instantly reduce the infectivity.

**Figure 6:**
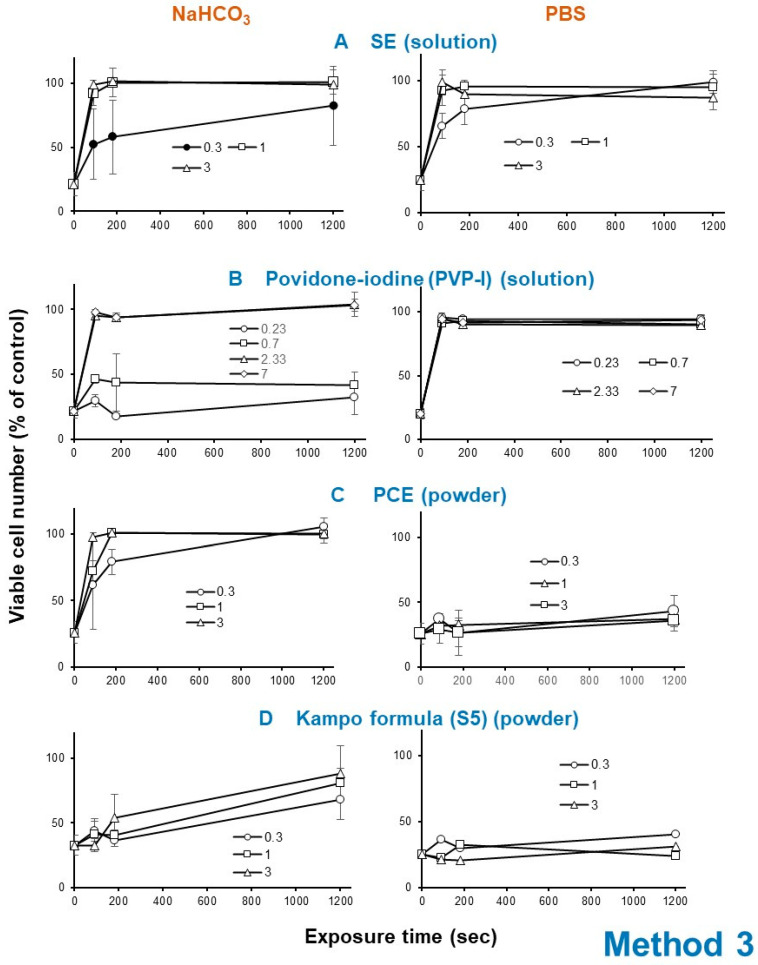
Effect of short exposure of HSV to SE (means of four independent experiments) (**A**), povidone iodine (**B**), PCE (**C**) and Kakkonto (S5) (**D**). A 100-fold higher titer of HSV was exposed to these samples for 0, 1.5, 3, or 20 min, and then added to the cells after dilution of 100-fold. After incubation for three days, viable cells were determined. Each value represents mean ± S.D. (n = 3).

Exposure of HSV with SE (A) (1 or 3 mg/mL), PCE (C) (1 or 3 mg/mL) as well as povidone iodine (B) (2.33 or 7 mg/mL) rapidly eliminated its infectivity within 3 min, whereas Kampo preparation (S5) (D) took 20 min to express HSV inactivation (Figure 6).

The HSV inactivation effect of SE was reproducibly diminished by dilution with 1.39% NaHCO_3_ (pH 8.0), rather than (PBS, pH 7.4) in four independent experiments (compare left and right column in Figure 6A). Povidone iodine showed similar instability under alkaline conditions (compare left and right column in Figure 6B). On the other hand, the anti-HSV activity of powders such as PCE and Kakkonto (S5) was enhanced more than 21.4 (=3/0.14) and 1.25-fold (=3/2.4), respectively, when they were first dissolved with 1.39% NaHCO_3_ rather than PBS (Figure 6C,D).

**Figure 7:**
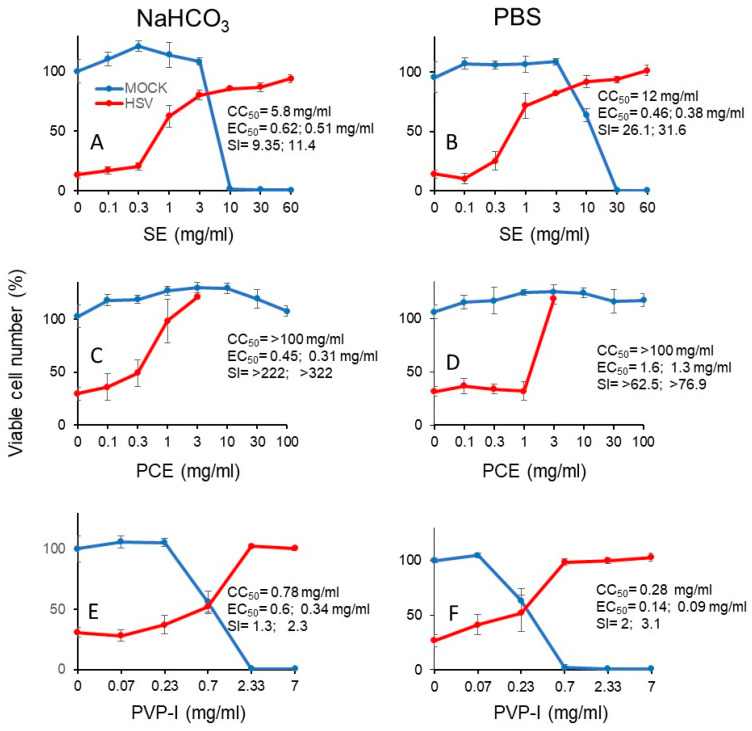
Dose-response curve of cytotoxicity and protective effect of short exposure (3 min) to SE (**A**, **B**), PCE (**C**,**D**), and PVP-I (**E**,**F**). Samples were dissolved and diluted either in 1.39% NaHCO_3_ or PBS. HSV and Vero cells were preincubated for 3 min, and chased into fresh medium, and the viable cell number was determined. The indicated concentrations in the abscissa is the concentration at the time of contact to samples for 3 min. Each value is represented as mean ± S.D. (n = 3).

We next investigated the cytotoxicity (measured by CC_50_) and protective effect (measured by EC_50_) of short exposure (3 min) of SE (A, B), PCA (C, D) and PV-I (E, F) (Figure 7). From the dose-response curve, we could calculate the SI values (Table 3). It is apparent that SE showed 2- to 3-fold higher anti-HSV activity when it was diluted with PBS (SI-I = 26.1, SI-II = 31.6) (B), rather than with NaHCO_3_ (SI-I = 9.4, SI-II = 11.4) (A). On the other hand, PCE showed higher anti-HSV activity when it was dissolved by NaHCO_3_ [SI-I > 222, SI-II > 322, MCR (maximum cell recovery) = 101.3%) (C), than by PBS (SI-I > 62.5, SI-II > 76.9, MCV = 26.8%) (D). However, we could not calculate the SI value of Kakkonto (S5) due to the lower protection effect. Povidone iodine (PVP-I) showed much lower anti-HSV activity, whenever diluted by NaHCO_3_ (SI-I = 1.3, SI-II = 2.3) (E) or PBS (SI-I = 2.0, SI-II = 3.1) (F) (Figure 7).

#### 3.3.2. Rapid HIV Inactivation by SE

**Figure 8:**
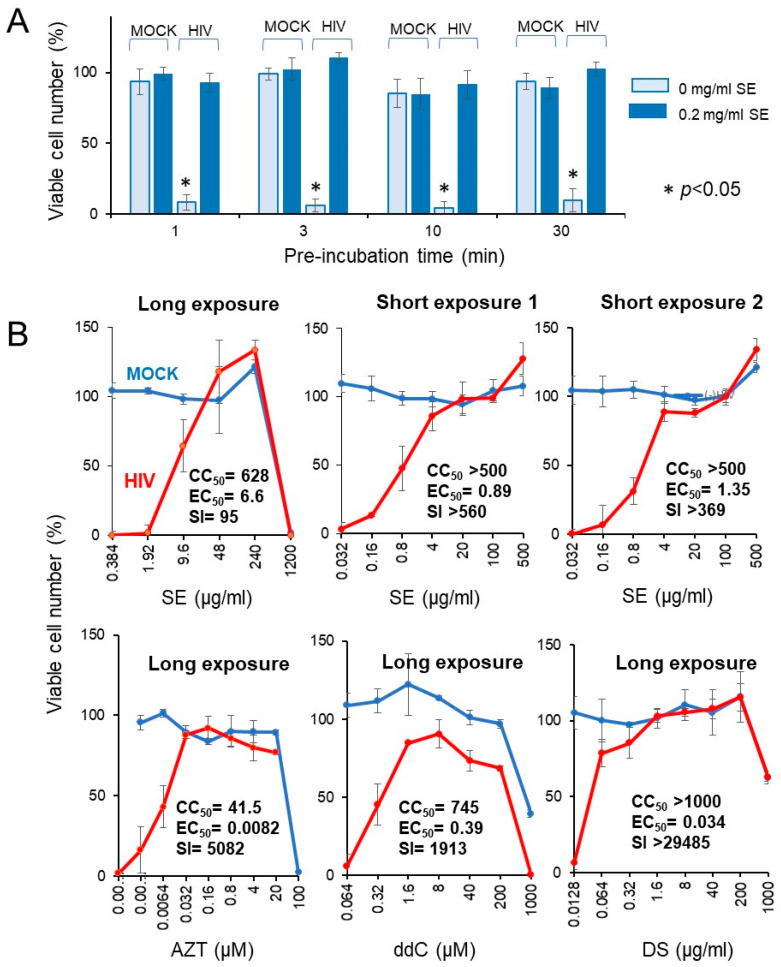
Rapid inactivation of HIV by SE. (**A**) Effect of preincubation time. A 20-fold higher titer of HIV (MOI = 0.2) was exposed to 0.2 mg/mL SE for 0, 1, 3, 10, or 30 min, and then added to the cells after dilution of 20-fold to make the final MOI = 0.01. After incubation for five days, viable cells were determined. Each value represented as the mean ± S.D. was determined (n = 3). HIV infection significantly reduced the viable cell number (*p* < 0.05). (**B**) Effect of long (five days) and short (10 min) exposure of HIV to SE, and popular anti-HIV agents on the cell viability. Each value represented as the mean ± S.D. was determined (n = 3).

We investigated whether the short exposure with SE enhanced the anti-HIV activity. We found that exposure of HIV to SE for as little as 1 ~ 30 min quickly inactivated the virus (Figure 8A). 

Four popular anti-HIV agents (AZT, ddC, DS, CRDS, used as the positive controls) showed potent anti-HIV activity (SI = 5082, 1913 > 29,485, 4666), verifying this system for measuring the anti-HIV activity (lower panel in Figure 8B). Compared with regular long exposure (five days) (SI = 95), short exposure to SE more effectively inactivated the virus (SI > 560, >369), yielding more than a 4 ~ 6-fold increase (upper panel in Figure 8B). We repeated the same experiment with more wider dose ranges, and found that 10 min exposure of HIV with SE showed approximately 20-fold increase of anti-HIV activity (25.1 and 16.6-fold in two different assays) when compared with regular longer exposure (Table 4).

## 4. Discussion

The present study demonstrated that the anti-HSV activity of the Kampo formula solely depended on the solubility. Kampo formulas are all powder, and therefore they have to be dissolved well and sterilized before treatment. We found that when they were directly dissolved with medium, no anti-HSV activity (Method 1) (Supplementary Table S1) and anti-HIV activity [36] were detected. On the other hand, when they were dissolved in alkaline solution such as 1.39% NaHCO_3_ (pH 8), some anti-HSV activity was recovered (Method 2). However, when they were dissolved with neutral buffer such as PBS (pH 7.4), no anti-HSV activity was recovered. Using Method 3, contact with Kakkonto for 20 min reduced the infectivity of HSV, consistent with previous reports of the anti-HSV activity of Kakkonto [12]. We recently reported that most Kampo formulas including Kakkonto showed protection against cisplatin and amyloid-β-induced neurotoxicity [36].

We have previously separated various polysaccharide fractions of the pine cone of *Pinus parviflora* Sieb et Zucc by successive hot water and alkaline extractions. Hot water extract contains Fr. I (neutral), Fr. II (uronic acid-rich), and Fr. V (tightly bound to diethylaminoethyl cellulose (DEAE) cellulose chromatography). Alkaline extract contains Fr.VI (acid-precipitable), and Fr. VII, VIII, and IX (step-wise precipitated by increasing amounts of ethanol). All of these fractions contain glucose, mannose, galactose, and arabinose or fucose as the main component of polysaccharide. Chemical analysis (infrared spectroscopy (IR), nuclear magnetic resonance (NMR), thin layer chromatography (TLC)) identified Frs. V, VI, VII, VIII, and IX as a lignin-carbohydrate complex [31]. We found that only Frs. V, VI, VII, VIII, and IX showed potent anti-HIV activity whereas neutral and acidic polysaccharides (Frs. I and II) were inactive [32]. It is reasonable that PCE was richer in lignified material than the Kampo formula, and showed higher anti-HSV activity. Removal of dextrin from PCE power may further increase the specific activity of anti-HSV.

SE itself is an alkaline solution containing a lignin-carbohydrate complex and its degradation product such as *p*-coumaric acid, which has no anti-HSV activity. We found that dilution of SE with 1.39% NaHCO_3_ reproducibly reduced the anti-HSV activity, possibly due to the degradation of the lignin-carbohydrate complex under alkaline condition. We reported diverse biological activity of SE (anti-inflammatory, antiviral, antibacterial, anti-UV, and anti-halitosis activity, and synergism with acyclovir or vitamin C), some of which overlapped that of the lignin-carbohydrate complex [13,16,17,29,32,37,38,39,40,41,42,43,44,45] (Table 5).

**Figure 9:**
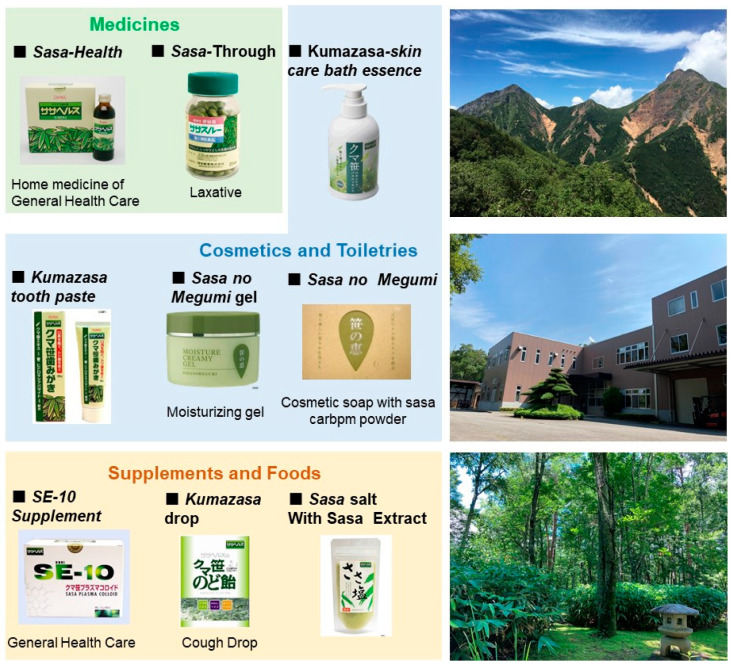
Medicines, cosmetics, toiletries, supplements, and foods manufactured from SE. Right column: Yatsugatake mountain peak (**top**), factory of the Daiwa Biological Research Institute Co. Ltd., Chino, Nagano, Japan (**middle**), and the botanical garden in the “*Sasa* Rikyu (imperial villa)” (**bottom**).

There are three commercially available products of alkaline extract of *Sasa* sp. (products A, B and C). SE (Product A) contains Fe (II)-chlorophyllin, whereas products B and C contain Cu (II)-chlorophyllin and less lignin-carbohydrate complex. Product C is supplemented with ginseng and pine (*Pinus densiflora*) leaf extracts. We found that SE (Product A) exhibited higher anti-HIV, anti-UV, and hydroxyl radical-scavenging activities compared to those of products B and C [46]. This finding further strengthens that major biological principles in SE may be the lignin-carbohydrate complex. The lignin-carbohydrate complex can be extracted by alkaline solution, but, once isolated by the alkaline solution, may be unstable under alkaline conditions [47,48,49], and gradually decompose into phenylpropanoid monomers and oligomers with little or no antiviral activity [50,51]. Based on these unique biological activities, we manufactured various medicines, cosmetics, toiletries, supplements, and foods using SE (Figure 9). If we could remove lower molecular weight degradation products that have essentially no antiviral activity, specific activity of SE may be further elevated. Considering that saliva is neutral with a pH of 7.2 ~ 7.3 [52] or 7.0 ~ 7.2 [53], SE may be stable in the oral cavity.

It was unexpected that lower molecular weight polyphenols such as resveratrol, curcumin, and *p*-coumaric acid had little or no anti-HSV activity. This may be due to their potent cytotoxicity against Vero cells. We have previously reported that tricin, but not the other four polymethoxyflavonoids (3,3’,4’,5,6,7,8-heptamethoxyflavone, nobiletin, tangeretin, and sudachitin), showed potent anti-HSV activity, suggesting the importance of the 3D-structure of these polymethoxyflavonoids for expressing anti-HISV activity [19]. It remains to be investigated whether short-term exposure of HSV to a higher concentration of these polyphenols may inactivate HSV or not.

We recently found that many chromone derivatives, esters, and amides showed much higher cytotoxicity against human oral squamous cell carcinoma cell lines when compared with human normal mesenchymal oral cells (gingival fibroblast, periodontal ligament fibroblast, pulp cells). Their tumor-specificity exceeded that of the lower molecular weight polyphenols. Furthermore, they showed much less normal keratinocyte toxicity than conventional anticancer drugs. Our preliminary study demonstrated that some of them alleviated the HSV-induced cytopathic effects. However, there was no correlation between their tumor-specificity and anti-HSV activity (Supplementary Table S3).

Recently, povidone iodine has been broadcasted to improve the symptoms of corona virus-infected patients in TV ASAHI super-channel in Japan on August 5, 2020. However, many authorities of medical sciences have shown cautionary stance, since it may kill the good bacteria that protect the mouth and reduce thyroid function. The present study showed that it also rapidly reduced the infectivity of HSV. Further study of the safety of this gargle as an antiviral agent should be performed.

We have previously demonstrated that ^125^I-labeled lignin-carbohydrate complex bound tightly to the influenza virus with sucrose gradient centrifugation [54]. The lignin-carbohydrate complex [55] and tannic acid [56] significantly inhibited the adsorption of ^3^H-labeled HSV to Vero cells [18]. Anti-HSV activity of these substances was much greater when they were added during virus adsorption to the cells rather than before and after adsorption [18,52]. These data suggest that the target of these substances may be virus or cell surface components. Since the lignin-carbohydrate complex significantly enhanced the expression of dectin-2 [53], possible interactions with the cell surface receptor should be investigated. Further study is necessary to identify the antiviral mechanisms of these substances.

## 5. Conclusions

The present study demonstrated for the first time that:

- Alkaline extracts of the leaves of *Sasa* sp. (SE) and pine cone extract (PCE) showed higher anti-HSV activity than 20 Japanese traditional herb medicines (Kampo formulas), resveratrol, *p*-coumaric acid, curcumin, tricin, and 119 chromone-related compounds. This confirms our previous finding that the alkaline extract of tea and licorice root showed higher anti-HIV activity than the respective hot water extract [57,58].
- Exposure of HSV to SE or PCE for 3 min almost completely eliminated the infectivity of HSV, whereas a much longer exposure time was required for Kakkonto, the most active Kampo formulae.
- Anti-HSV activity of PCE and Kakkonto could be detected only when they were dissolved by an alkaline solution (pH 8.0), but not by neutral buffer (pH 7.4).
- Anti-HSV activity of SE and povidone iodine was unstable if they were diluted with alkaline solution.
- Anti-HSV activity of SE and PCE were one or two-orders higher than povidone iodide.
- Anti-HIV activity of SE was also enhanced when it was administered for a short period.
- The present study suggests the applicability of a short treatment of oral virus with SE and PCE.

## Figures and Tables

**Table 1:** Twenty Kampo formulas used in this study.

S1	Unkeito (TJ-106)	S11	Jumihaidokuto (TJ-6)
S2	Chotosan (TJ-47)	S12	Yokuininto (TJ-52)
S3	Hochuekkito (TJ-41)	S13	Shofusan (TJ-22)
S4	Hangebyakujutsutemmato (TJ-37)	S14	Hainosankyuto (TJ-122)
S5	Kakkonto (TJ-1)	S15	Jizusoippo (TJ-59)
S6	Shomakakkonto (TJ-101)	S16	Unseiin (TJ-57)
S7	Sokeikakketsuto (TJ-53)	S17	Rikkosan (TJ-110)
S8	Seijobofuto (TJ-58)	S18	Keigairengyoto (TJ-50)
S9	Yokukansan (TJ-54)	S19	Sansoninto (TJ-103)
S10	Orengedokuto (TJ-15)	S20	Kakkontokasenkyushin’I (TJ-2)

**Table 2:** Quantification of anti-HSV activity of Kampo formulas with reference compounds.

Test Sample	Viability of HSV- Infected Cells (%)	CC_50_	EC_50_-I	EC_50_-II	Anti- HSV Activity		Max. Cell Recovery (%)
					SI-I	SI-II	
Kampo formula (Supplementary Figure S2) (mg/mL)							
S1 Unkeito	27	>3.0	(-)	1	(-)	>3.0	51
S2 Chotosan	25	2.6	(-)	>3.0	(-)	<0.87	48
S3 Hochuekkito	25	>3.0	(-)	>3.0	(-)	><1.0	44
S4 Hangebyakujutsutemmato	26	1.35	(-)	>3.0	(-)	<0.45	41
S5 Kakkonto	29	1.65	0.58	0.56	**>5.2**	**>5.4**	**78**
S6 Shomakakkonto	34	2.1	(-)	>3.0	(-)	<0.70	46
S7 Sokeikakketsuto	29	>3.0	(-)	>3.0	(-)	><1.0	46
S8 Seijobofuto	34	0.58	(-)	>3.0	(-)	<0.19	39
S9 Yokukansan	31	>3.0	2.9	1.9	>1.0	>1.6	**66**
S10 Orengedokuto	22	0.76	(-)	>3.0	(-)	<0.25	30
S11 Jumihaidokuto	25	3	(-)	2.8	(-)	1.1	**52**
S12 Yokuininto	21	>3.0	(-)	2.4	(-)	1.3	**55**
S13 Shofusan	21	2.5	(-)	>3.0	(-)	<0.83	34
S14 Hainosankyuto	24	2.6	(-)	>3.0	(-)	<0.87	33
S15 Jizusoippo	23	1.3	(-)	>3.0	(-)	<0.43	33
S16 Unseiin	23	1.5	(-)	>3.0	(-)	<0.5	29
S17 Rikkosan	21	1.8	(-)	>3.0	(-)	<0.60	34
S18 Keigairengyoto	26	1.6	(-)	>3.0	(-)	<0.53	34
S19 Sansoninto	23	1.6	(-)	>3.0	(-)	<0.53	47
S20 Kakkontokasenkyushin’I	32	2	(-)	>3.0	(-)	<0.67	45
Alkaline extracts (mg/mL)							
SE (Supplementary Table S1)	20	2.6	0.7	0.5	**4.5**	**6.8**	**90**
PCE	19	2	0.19	0.17	**13.1**	**14.7**	**82**
**Polyphenols** (µM)							
Resveratrol	16	18	(-)	>1000	(-)	<0.018	20
*p*-Coumaric acid	42	170	(-)	>1000	(-)	<0.17	43
Curcumin	22	16	(-)	>100	(-)	<0.16	42
Chromones (µM) (Supplementary Table S2)							
(2a) (Ref. 20)	9	>600	450	400	**>1.3**	>1.5	**59**
(3c) (Ref. 20)	9	310	NT	300	NT	1.0	50
(14) (Ref. 21)	11	31	NT	29	NT	1.1	**51**
(2) (Ref. 24)	11	>1000	820	640	>1.2	>1.6	**61**
(8) (Ref. 25)	23	86	ND	180	ND	**>3.3**	**54**
(12) (Ref. 25)	23	>600	ND	370	ND	**>1.6**	**64**
Positive controls (µM)							
ACV	23	>30	1.3	1.1	**>23.1**	**>27.3**	**108**
Tricin	18	10	(-)	1.4	(-)	**7.1**	**68**

Data were derived from Supplementary Figure S2 (for all 20 Kampo formula) and from Figure 4. (2a), 2-(1*H*-pyrazol-1-yl)-4*H*-1-benzopyran-4-one; (3c), 2-(1*H*-imidazol-1-yl)-6-methoxy-4*H*-1-benzopyran-4-one; (14), (3*E*)-2,3-dihydro-3-[(4-hydroxyphenyl)methylene]-7-methoxy-4*H*-1-benzopyran-4-one; (2), (2*E*,4*E*)-5-(3,4-methylenedioxyphenyl)-2,4-pentadienoic acid (4-hydroxy-3-methoxyphenyl)methyl ester; (8), 2-[(1*E*)-2-(4-fluorophenyl)ethenyl]-6-methoxy-4*H*-1-benzopyran-4-one; (12), 2-[(1*E*)-2-(3,4-dimethoxy) ethenyl]-6-methoxy-4*H*-1-benzopyran-4-one. Supplementary Tables S2 and S3 show the anti-HSV activity of SE (assayed 52 times) and a total of 119 chromone derivatives, esters, and amides.

**Table 3:** Quantification of HSV inactivation by short exposure to SE and PCE.

	Exposure Time (min)		Viability of HSV- Infected Cells (%)	CC_50_ (mg/mL)	EC_50_-I (mg/mL)	EC_50_-II (mg/mL)	Anti-HSV Activity		Max. Cell Recovery (%)
							SI-I	SI-II	
SE	3	NaHCO_3_	21.3	5.8	0.62	0.51	9.4	11.4	101.7
(solution)	**3**	PBS	24.8	12.0	0.46	0.38	26.1	31.6	98.8
PCE	**3**	NaHCO_3_	25.9	>100	0.45	0.31	>222	>322	101.3
(powder)	3	PBS	25.9	>100	1.6	1.3	>62.5	>76.9	26.8
S5	3	NaHCO_3_	32.7	>100	(-)	(-)	(-)	(-)	88.3
(powder)	3	PBS	25.0	>100	(-)	(-)	(-)	(-)	40.6
PVP-I	3	NaHCO_3_	21.8	0.78	0.6	0.34	1.3	2.3	103.1
(solution)	3	PBS	20..2	0.28	0.14	0.09	2.0	3.1	94.3

**Table 4:** Enhancement of anti-HIV activity of SE by shortening the treatment time.

	CC_50_ (µg/mL)	EC_50_ (µg/mL)	SI	n-Fold
SE (long exposure)	627.80	6.63	95	1
SE (short exposure 1)	>500	0.894	>560	>5.9
Repeat	1067	0.447	2388	25.1
SE (short exposure 2)	>500	1.35	>369	>3.9
Repeat	1067	0.677	1577	16.6
Positive controls				
AZT (µM)	41.49	0.00817	5082	
ddC (µM)	745.32	0.390	1913	
DS	>1000	0.0339	>29485	
CRDS	704.96	0.151	4666	

**Table 5:** Diverse biological activity of SE and similarity with the lignin-carbohydrate complex.

Biological Activity	SE	Lignin–Carbohydrate Complex
Anti-inflammatory activity	[37,38]	[32]
Antiviral activity	[29]	[16,32]
Antibacterial activity	[29,39]	
Anti-UV activity	[40]	[41,42]
Synergism with acyclovir (Antiviral)	[13]	
Synergism with vitamin C (Anti-UV)	[43]	
Synergism with vitamin C (antitumor)		[44]
Anti-halitosis activity	[45]	

## Supplementary Materials

The following are available online at https://www.mdpi.com/2305-6320/7/10/64/s1, Figure S1: Method I. Direct mixing of Kampo preparations with culture medium resulted in low recovery of anti-HSV activity, Figure S2: Method 2: Weak anti-HSV activity was detected in S5, S9, S11 and S12, Table S1: Higher anti-HSV activity of Kampo formulas was recovered by dissolving with 1.39% NaHCO_3_ than with PBS, Table S2. Anti-HSV activity of SE from 52 experiments, Table S3. Anti-HSV activity of chromones, esters, and amides (119 compounds).
