# Supplementary material for: Quantification of the Ability of Natural Products to Prevent Herpes Virus Infection
Source: Medicines (Basel). 2020 Oct 6;7(10):64. doi: 10.3390/medicines7100064 (PMC7601274; doi:10.3390/medicines7100064)
Supplement: Supplementary file 1 [file medicines-07-00064-s001.pdf]

Supplementary Materials: Quantification of the Ability of Natural Products to Prevent Herpes Virus Infection

Kunihiko Fukuchi, Hiroshi Sakagami, Yoshiaki Sugita, Koichi Takao, Daisuke Asai, Shigemi Terakubo, Hiromu Takemura, Hirokazu Ohno, Misaki Horiuchi, Madoka Suguro, Tomohiro Fujisawa, Kazuki Toeda, Hiroshi Oizumi, Toshikazu Yasui and Takaaki Oizumi

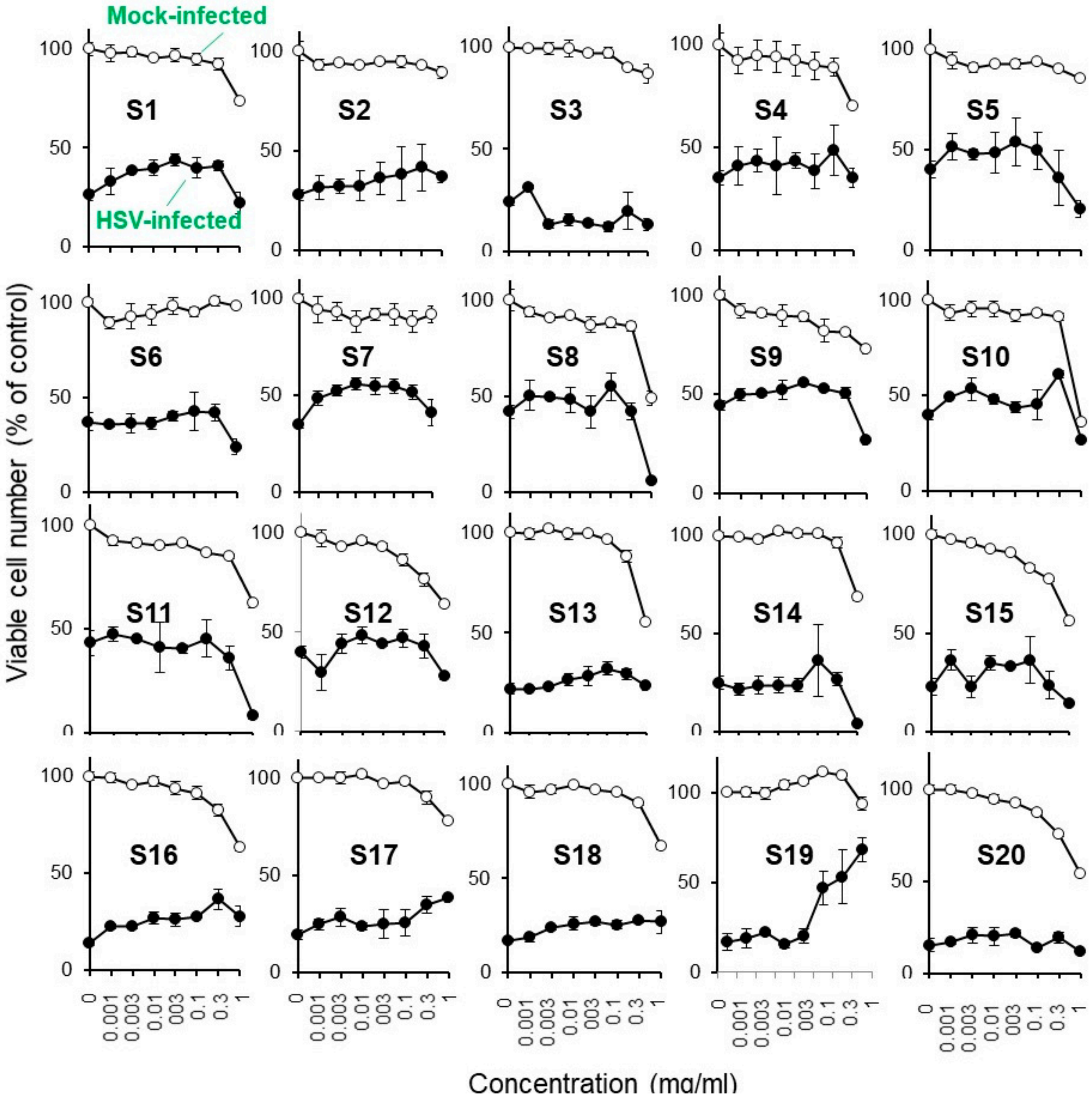

Figure S1. Method 1. Direct mixing of Kampo preparations with culture medium resulted in low recovery of anti-HSV activity.

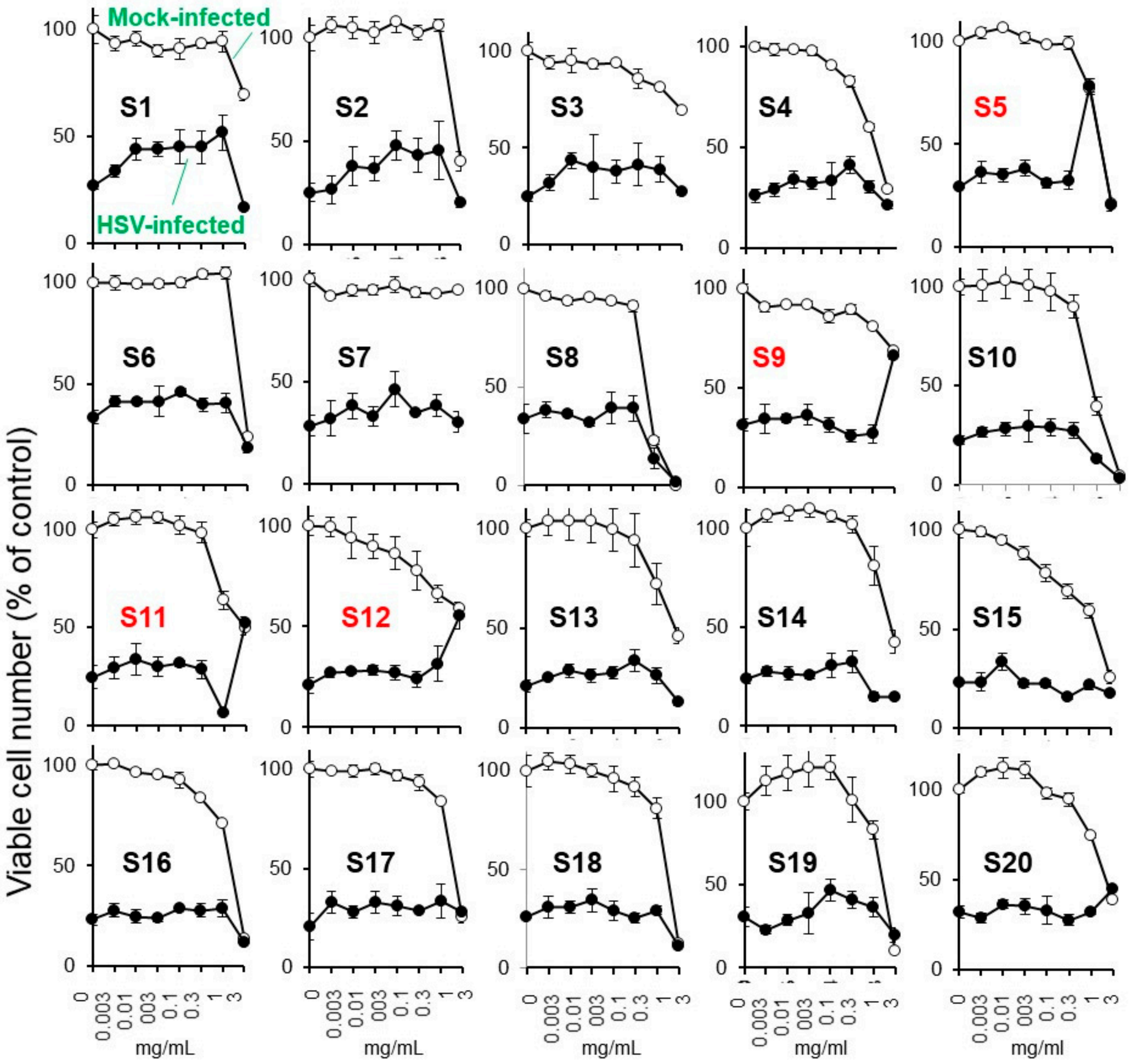

Figure S2 Method 2: Weak anti-HSV activity was detected in S5, S9, S11 and S12

Table S1. Higher anti-HSV activity of Kampo formulas was recovered by dissolving with 1.39% $NaHCO_3$ than with PBS.

			Dissolved with	EC-1 (mg/ml)	ECII (mg/ml)	SI I	SI II	Max. cell Recovery (%)	CC_{50} (mg/ml)	EC-1 (mg/ml)	ECII (mg/ml)	Max. cell Recovery (%)
S1	Unkeito	Method 1		(-)	(-)	(-)	(-)	(-)				
		Method 2	$NaHCO_3$	(-)	1	(-)	(-)	51	>3			
		Method 3	$NaHCO_3$	(-)	(-)	NA	NA	(-)				
		Method 3	PBS	(-)	(-)	NA	NA	(-)				
S2	Chotosan	Method 1		(-)	(-)	(-)	(-)	(-)				
		Method 2	$NaHCO_3$	(-)	(-)	(-)	(-)	(-)	2.6			
		Method 3	$NaHCO_3$	(-)	(-)	NA	NA	(-)				
		Method 3	PBS	(-)	(-)	NA	NA	(-)				
S3	Hochuekkito	Method 1		(-)	(-)	(-)	(-)	(-)				
		Method 2	$NaHCO_3$	(-)	(-)	(-)	(-)	(-)	>3			
		Method 3	$NaHCO_3$	(-)	(-)	NA	NA	(-)				
		Method 3	PBS	(-)	(-)	NA	NA	(-)				
S4	Hangebyakujutsutemmato	Method 1		(-)	(-)	(-)	(-)	(-)				
		Method 2	$NaHCO_3$	(-)	(-)	(-)	(-)	(-)	1.35			
		Method 3	$NaHCO_3$	(-)	(-)	NA	NA	(-)				
		Method 3	PBS	(-)	(-)	NA	NA	(-)				
S5	Kakkonto	Method 1		(-)	(-)	(-)	(-)	(-)				
		Method 2	$NaHCO_3$	0.58	0.56	>5.2	>5.4	91	1.65			
		Method 3	$NaHCO_3$	0.5	0.46	NA	NA	101		0.37	0.34	101
		Method 3	PBS	(-)	(-)	NA	NA	(-)				
S6	Shomakakkonto	Method 1		(-)	(-)	(-)	(-)	(-)				
		Method 2	$NaHCO_3$	(-)	(-)	(-)	(-)	(-)	2.1			
		Method 3	$NaHCO_3$	>3	>3	NA	NA	80		2.6	0.18	85
		Method 3	PBS	(-)	(-)	NA	NA	(-)				
S7	Sokeikakketsuto	Method 1		(-)	(-)	(-)	(-)	(-)				
		Method 2	$NaHCO_3$	(-)	(-)	(-)	(-)	(-)	>3			
		Method 3	$NaHCO_3$	(-)	(-)	NA	NA	(-)		(-)	(-)	(-)
		Method 3	PBS	(-)	(-)	NA	NA	(-)				
S8	Seijobofuto	Method 1		(-)	(-)	(-)	(-)	(-)				
		Method 2	$NaHCO_3$	(-)	(-)	(-)	(-)	(-)	0.58			
		Method 3	$NaHCO_3$	>3	>3	NA	NA	90		12	7.2	112
		Method 3	PBS	6.6	52	NA	NA	77		12	8.8	100
S9	Yokukansan	Method 1		(-)	(-)	(-)	(-)	(-)				
		Method 2	$NaHCO_3$	2.9	1.9	>1	>1.6	66	>3			
		Method 3	$NaHCO_3$	>3	>3	NA	NA	76		0.42	0.085	104
		Method 3	PBS	(-)	(-)	NA	NA	(-)				
S10	Orengedokuto	Method 1		(-)	(-)	(-)	(-)	(-)				
		Method 2	$NaHCO_3$	(-)	(-)	(-)	(-)	(-)	0.76			
		Method 3	$NaHCO_3$	8	3	NA	NA	68		(-)	(-)	(-)
		Method 3	PBS	9.6	6.2	NA	NA	60				
S11	Jumihaidokuto	Method 1		(-)	(-)	(-)	(-)	(-)				
		Method 2	$NaHCO_3$	(-)	2.8	(-)	1.1	52	3			
		Method 3	$NaHCO_3$	>3	>3	NA	NA	94		0.36	0.28	105
		Method 3	PBS	23	15	NA	NA	86		50	20	59
S12	Yokuininto	Method 1		(-)	(-)	(-)	(-)	(-)				
		Method 2	$NaHCO_3$	(-)	2.4	(-)	1.3	55	>3			
		Method 3	$NaHCO_3$	>3	>3	NA	NA	90		0.4	0.29	90
		Method 3	PBS	5.2	3.6	NA	NA	74		(-)	(-)	(-)
S13	Shofusan	Method 1		(-)	(-)	(-)	(-)	(-)				
		Method 2	$NaHCO_3$	(-)	(-)	(-)	(-)	(-)	2.5			
		Method 3	$NaHCO_3$	(-)	(-)	NA	NA	(-)				
		Method 3	PBS	(-)	(-)	NA	NA	(-)				
S14	Hainosankyuto	Method 1		(-)	(-)	(-)	(-)	(-)				
		Method 2	$NaHCO_3$	(-)	(-)	(-)	(-)	(-)	2.6			
		Method 3	$NaHCO_3$	12	<3	NA	NA	98		11	8.4	93
		Method 3	PBS	27	20	NA	NA	90		44	19	74
S15	Jizusoippo	Method 1		(-)	(-)	(-)	(-)	(-)				
		Method 2	$NaHCO_3$	(-)	(-)	(-)	(-)	(-)	1.3			
		Method 3	$NaHCO_3$	<3	<3	NA	NA	99		1.3	1.1	71
		Method 3	PBS	(-)	(-)	NA	NA	(-)				
S16	Unseiin	Method 1		(-)	(-)	(-)	(-)	(-)				
		Method 2	$NaHCO_3$	(-)	(-)	(-)	(-)	(-)	1.5			
		Method 3	$NaHCO_3$	23	18	NA	NA	66.2		(-)	(-)	(-)
		Method 3	PBS	(-)	(-)	NA	NA	(-)				
S17	Rikkosan	Method 1		(-)	(-)	(-)	(-)	(-)				
		Method 2	$NaHCO_3$	(-)	(-)	(-)	(-)	(-)	1.8			
		Method 3	$NaHCO_3$	16	10	NA	NA	103		45	40	87
		Method 3	PBS	56	47	NA	NA	87		34	7.4	98
S18	Keigairengyoto	Method 1		(-)	(-)	(-)	(-)	(-)				
		Method 2	$NaHCO_3$	(-)	(-)	(-)	(-)	(-)	1.6			
		Method 3	$NaHCO_3$	<3	<3	NA	NA	93.8		1.6	0.62	88
		Method 3	PBS	45	35	NA	NA	83.4		25	21	107
S19	Sansoninto	Method 1		0.45	0.17	>2.2	>5.9	68				
		Method 2	$NaHCO_3$	(-)	(-)	(-)	(-)	(-)	1.6			
		Method 3	$NaHCO_3$	14	17	NA	NA	100		8.4	6	127
		Method 3	PBS	37	27	NA	NA	82		13	11	92
S20	Kakkontokasenkyushin'I	Method 1		(-)	(-)	(-)	(-)	(-)				
		Method 2	$NaHCO_3$	(-)	(-)	(-)	(-)	(-)	2			
		Method 3	$NaHCO_3$	>3	>3	NA	NA	89		0.4	0.27	83
		Method 3	PBS	(-)	(-)	NA	NA	(-)				

Gray colors show Results of screening

Table S2. Anti-HSV activity of SE from 52 experiments.

Exp. No	Viability of HSV-infected cells (%)	CC_{50} (μM)	EC_{50}-I (μM)	EC_{50}-II (μM)	Anti-HSV activity SI-I	Anti-HSV activity SI-II	Max. cell recovery (%)
1	38.0	2.5	0.54	0.33	4.7	7.6	93.0
2	25.2	4.5	0.52	0.44	8.6	10.2	100.0
3	36.1	2	0.56	0.44	3.6	4.5	100.0
4	50.0	3	0.76	0.48	3.9	6.3	89.0
5	28.9	5	0.54	0.44	9.5	11.4	100.0
6	42.1	3.4	0.75	0.4	4.5	8.5	79.0
7	24.2	2.7	0.96	0.74	2.8	3.6	64.0
8	25.0	3.3	0.68	0.55	4.9	60.0	89.0
9	5.2	1.9	0.66	0.62	2.9	3.1	73.9
10	8.0	2.1	0.52	0.5	4.0	4.2	100.0
11	0.0	1.4	0.87	0.87	1.6	1.6	68.0
12	26.4	2	0.7	0.54	2.9	3.7	82.4
13	13.1	2	0.58	0.52	3.4	3.8	86.3
14	42.9	3.2	0.71	0.45	4.5	6.7	86.6
15	40.2	2.3	0.67	0.25	3.4	9.2	78.0
16	37.0	2.4	0.68	0.22	3.5	10.9	76.8
17	24.8	2.9	0.65	0.4	4.5	7.3	80.5
18	20.4	1.8	0.6	0.46	3.0	6.0	82.4
19	18.4	2.5	0.82	0.62	3.0	4.0	67.0
20	32.7	3	0.92	0.52	3.3	5.8	70.0
21	21.1	3	0.72	0.6	4.2	6.0	78.2
22	22.1	3	0.59	0.48	5.1	6.3	90.4
23	31.3	3	0.64	0.4	4.7	7.5	81.9
24	43.4	3	0.58	0.45	5.2	6.7	94.7
25	39.7	3	0.79	0.48	3.8	7.9	80.2
26	21.4	1.7	0.7	0.52	2.4	3.3	74.0
27	23.0	1.5	0.8	0.62	1.9	2.4	70.6
28	25.4	1.6	0.75	0.56	2.1	2.9	71.4
29	17.9	3	0.51	0.46	5.9	6.5	110.0
30	20.0	3	0.58	0.47	5.2	6.4	83.9
31	11.0	1.7	0.66	0.6	2.6	2.8	75.7
32	7.8	2	0.58	0.54	3.4	3.7	86.7
33	7.7	2	0.36	0.31	5.6	6.5	74.7
34	19.6	2.6	0.56	0.45	4.6	5.8	86.0
35	9.5	2.9	0.5	0.46	5.8	6.3	109.5
36	8.6	1.6	0.56	0.52	2.9	3.1	92.9
37	20.1	3.1	0.69	0.5	4.5	6.2	95.3
38	10.0	3	0.58	0.54	5.2	5.6	91.9
39	22.7	3	0.6	0.5	5.0	6.0	95.0
40	7.6	3.5	0.56	0.54	6.3	6.5	101.0
41	6.6	3	0.43	0.4	7.0	7.5	112.0
42	8.6	3	0.58	0.54	5.2	5.6	95.0
43	14.3	2.2	0.6	0.54	3.7	4.1	91.0
44	7.7	2.2	0.56	0.54	3.9	4.1	94.7
45	9.9	2.3	0.5	0.46	4.6	5.0	104.2
46	5.7	3	4.8	0.47	6.3	6.4	124.0
47	9.8	2.3	0.52	0.5	4.4	4.6	107.0
48	6.6	3	0.54	0.52	5.6	5.8	105.0
49	18.2	3	0.5	0.45	6.0	6.7	114.0
50	16.2	3	0.54	0.48	5.6	6.3	108.0
51	17.9	2.7	0.52	0.46	5.2	5.9	106.0
52	9.8	2.8	0.45	0.41	6.2	6.8	113.0
**mean**	**20.4**	**2.6**	**0.70**	**0.49**	**4.5**	**6.8**	**90.1**

Table S3. Anti-HSV activity of chromones, esters, and amides (119 compounds).

	TS (D/B)	CC_{50} against OSCC	CC_{50} against Vero	Viability of HSV-infected cells (%)	Range tested (μM)	EC_{50}-I (μM)	EC_{50}-II (μM)	SI-I	SI-II	Max. cell recovery (%)
2-(1*H*-pyrrol-1-yl)-4*H*-1-benzopyran-4-one [1a]	1.0	259.5	130	7	18-600	(-)	(-)	(-)	(-)	35
7-methoxy-2-(1*H*-pyrrol-1-yl)-4*H*-1-benzopyran-4-one [1b]	1.8	159.7	72	9	30-600	(-)	(-)	(-)	(-)	(-)
6-methoxy-2-(1*H*-pyrrol-1-yl)-4*H*-1-benzopyran-4-one [1c]	<0.9	>295	180	9	30-600	(-)	(-)	(-)	(-)	40
**2-(1*H*-pyrazol-1-yl)-4*H*-1-benzopyran-4-one [2a]**	**><1.0**	>400	>600	9	30-600	450	400	**>1.3**	**>1.5**	**59**
7-methoxy-2-(1*H*-pyrazol-1-yl)-4*H*-1-benzopyran-4-one [2b]	<1.0	>293	140	9	30-600	(-)	(-)	(-)	(-)	(-)
6-methoxy-2-(1*H*-pyrazol-1-yl)-4*H*-1-benzopyran-4-one [2c]	><1.0	>380	240	9	30-600	(-)	(-)	(-)	(-)	37
2-(1*H*-imidazol-1-yl)-4*H*-1-benzopyran-4-one [3a]	>5.4	71	160	9	30-600	(-)	(-)	(-)	(-)	25
2-(1*H*-imidazol-1-yl)-7-methoxy-4*H*-1-benzopyran-4-one [3b]	><1.0	>400	270	9	30-600	(-)	(-)	(-)	(-)	21
**2-(1*H*-imidazol-1-yl)-6-methoxy-4*H*-1-benzopyran-4-one [3c]**	**><0.7**	>400	310	9	30-600	NT	300	NT	**1**	**50**
2-(1*H*-1,2,4-triazol-1-yl)-4*H*-1-benzopyran-4-one [4a],	>1.7	153.5	180	9	30-600	(-)	(-)	(-)	(-)	42
7-methoxy-2-(1*H*-1,2,4-triazol-1-yl)-4*H*-1-benzopyran-4-one [4b]	>2.2	105.7	140	9	30-600	(-)	(-)	(-)	(-)	30
6-methoxy-2-(1*H*-1,2,4-triazol-1-yl)-4*H*-1-benzopyran-4-one [4c]	><0.9	>244	120	15	30-600	(-)	(-)	(-)	(-)	30
2-(1*H*-1,2,3-triazol-1-yl)-4*H*-1-benzopyran-4-one [5a]	>5.0	50	120	15	30-600	(-)	(-)	(-)	(-)	39
7-methoxy-2-(1*H*-1,2,3-triazol-1-yl)-4*H*-1-benzopyran-4-one [5b]	>4.6	54.7	110	15	30-600	(-)	(-)	(-)	(-)	39
6-methoxy-2-(1*H*-1,2,3-triazol-1-yl)-4*H*-1-benzopyran-4-one [5c]	>4.7	46.6	90	15	30-600	(-)	(-)	(-)	(-)	38
2-(1*H*-indol-1-yl)-4*H*-1-benzopyran-4-one [6a]	**>37.5**	6.1	14	15	3-100	(-)	(-)	(-)	(-)	(-)
2-(1*H*-indol-1-yl)-7-methoxy-4*H*-1-benzopyran-4-one [6b]	**24.2**	6.3	6.3	15	3-100	(-)	(-)	(-)	(-)	16
2-(1*H*-indol-1-yl)-6-methoxy-4*H*-1-benzopyran-4-one [6c]	**24.1**	1.5	6	15	3-100	(-)	(-)	(-)	(-)	11
2-(1*H*-indazol-1-yl)-4*H*-1-benzopyran-4-one [7a]	>7.8	51	40	15	30-600	(-)	(-)	(-)	(-)	(-)
2-(1*H*-indazol-1-yl)-7-methoxy-4*H*-1-benzopyran-4-one [7b]	>5.3	72.2	120	15	30-600	(-)	(-)	(-)	(-)	(-)
2-(1*H*-indazol-1-yl)-6-methoxy-4*H*-1-benzopyran-4-one [7c]	><1.1	>214	45	15	30-600	(-)	(-)	(-)	(-)	(-)
2-(1*H*-benzimidazol-1-yl)-4*H*-1-benzopyran-4-one [8a]	3.5	74.9	55	7.7	30-600	(-)	(-)	(-)	(-)	(-)
2-(1*H*-benzimidazol-1-yl)-7-methoxy-4*H*-1-benzopyran-4-one [8b]	>9.5	22.7	110	7.7	30-600	(-)	(-)	(-)	(-)	14
2-(1*H*-benzimidazol-1-yl)-6-methoxy-4*H*-1-benzopyran-4-one [8c]	13.0	4.7	13	7.7	3-600	(-)	(-)	(-)	(-)	(-)
(3*E*)-2,3-Dihydro-3-(phenylmetylene)-4*H*-1-benzopyran-4-one [1]	8.1	9.4	16	10	3-100	(-)	(-)	(-)	(-)	11
(3*E*)-2,3-dihydro-3-[(4-hydroxyphenyl)methylene]-4*H*-1-benzopyran-4-one [2]	13.2	28.3	19	10	3-100	(-)	(-)	(-)	(-)	22
(3*E*)-2,3-dihydro-3-[(3,4-dihydroxyphenyl)methylene]-4*H*-1-benzopyran-4-one [3]	>22.4	13.6	15	10	3-100	(-)	(-)	(-)	(-)	16
(3*E*)-2,3-dihydro-3-[(4-methoxyphenyl)methylene]-4*H*-1-benzopyran-4-one [4]	8.7	15.1	18	10	3-100	(-)	(-)	(-)	(-)	16
(3*E*)-2,3-dihydro-3-[(3,4-dimethoxyphenyl)methylene]-4*H*-1-benzopyran-4-one [5]	8.9	7.8	14	10	3-100	(-)	(-)	(-)	(-)	15
(3*E*)-2,3-dihydro-3-[(4-dimethylaminophenyl)methylene]-4*H*-1-benzopyran-4-one [6]	>20.3	19.7	34	10	3-100	(-)	(-)	(-)	(-)	17
(3*E*)-2,3-dihydro-3-[(4-fluorophenyl)methylene]-4*H*-1-benzopyran-4-one [7]	14.9	8.3	14	10	3-100	(-)	(-)	(-)	(-)	15
(3*E*)-3-[(4-chlorophenyl)methylene]-2,3-dihydro-4*H*-1-benzopyran-4-one [8]	>28.1	14	22	10	3-100	(-)	(-)	(-)	(-)	10
(3*E*)-2,3-dihydro-7-hydroxy-3-[(4-hydroxyphenyl)methylene]-4*H*-1-benzopyran-4-one [9]	7.3	27.2	15	11	10-300	(-)	(-)	(-)	(-)	18
(3*E*)-2,3-dihydro-3-[(3,4-dihydroxyphenyl)methylene]-7-hydroxy-4*H*-1-benzopyran-4-one [10]	>24.2	16.5	18	11	10-300	(-)	(-)	(-)	(-)	49
(3*E*)-2,3-dihydro-7-hydroxy-3-[(4-methoxyphenyl)methylene]-4*H*-1-benzopyran-4-one [11]	6.8	25.5	20	11	10-300	(-)	(-)	(-)	(-)	33
(3*E*)-2,3-dihydro-3-[(4-dimethylaminophenyl)methylene]-7-hydroxy-4*H*-1-benzopyran-4-one [12]	**35.4**	4.8	9	11	0.3-10	(-)	(-)	(-)	(-)	(-)
(3*E*)-2,3-dihydro-7-methoxy-3-(phenylmethylene)-4*H*-1-benzopyran-4-one [13]	8.3	10.7	17	11	10-300	(-)	(-)	(-)	(-)	12
**(3*E*)-2,3-dihydro-3-[(4-hydroxyphenyl)methylene]-7-methoxy-4*H*-1-benzopyran-4-one [14]**	**6.2**	25.6	31	11	10-300	NT	29	NT	**1.1**	**51**
(3*E*)-2,3-dihydro-3-[(3,4-dihydroxyphenyl)methylene]-7-methoxy-4*H*-1-benzopyran-4-one [15]	**>55.2**	7.3	25	11	3-100	(-)	(-)	(-)	(-)	46
(3*E*)-2,3-dihydro-7-methoxy-3-[(4-methoxyphenyl)methylene]-4*H*-1-benzopyran-4-one [16]	>22.2	11.6	24	11	3-100	(-)	(-)	(-)	(-)	35
(3*E*)-2,3-dihydro-3-[(4-dimethylaminophenyl)methylene]-7-methoxy-4*H*-1-benzopyran-4-one [17]	1.2	>327	>300	11	10-300	(-)	(-)	(-)	(-)	25
(2*E*)-1-(2-Hydroxyphenyl)-3-phenyl-2-propen-1-one (1)	2.5	24.8	21	8	3-100	(-)	(-)	(-)	(-)	12
(2*E*)-1-(2-hydroxyphenyl)-3-(4-hydroxyphenyl)-2-propen-1-one (2)	4.6	41.1	53	8	3-100	(-)	(-)	(-)	(-)	11
(2*E*)-1-(2-hydroxyphenyl)-3-(4-methoxyphenyl)-2-propen-1-one (3)	3.0	30.2	41	8	3-100	(-)	(-)	(-)	(-)	(-)
(2*E*)-3-(3,4-dimethoxyphenyl)-1-(2-hydroxyphenyl)-2-propen-1-one (4)	3.4	34.2	36	8	3-100	(-)	(-)	(-)	(-)	11
(2*E*)-3-(4-fluorophenyl)-1-(2-hydroxyphenyl)-2-propen-1-one (5)	2.6	19.3	25	8	3-100	(-)	(-)	(-)	(-)	(-)
(2*E*)-3-(4-chlorophenyl)-1-(2-hydroxyphenyl)-2-propen-1-one (6)	2.4	12.1	14	8	3-100	(-)	(-)	(-)	(-)	(-)
(2*E*)-3-(4-bromophenyl)-1-(2-hydroxyphenyl)-2-propen-1-one (7)	3.3	10.9	8.4	7	3-100	(-)	(-)	(-)	(-)	11
(2*E*)-1-(2-hydroxy-4-methoxyphenyl)-3-(4-hydroxyphenyl)-2-propen-1-one (8)	3.7	33.6	23	7	3-100	(-)	(-)	(-)	(-)	26
(2*E*)-1-(2-hydroxy-4-methoxyphenyl)-3-phenyl-2-propen-1-one (9)	3.5	13	15	7	3-100	(-)	(-)	(-)	(-)	14
(2*E*)-1-(2-hydroxy-4-methoxyphenyl)-3-(4-methoxyphenyl)-2-propen-1-one (10)	6.0	26.6	26	7	3-100	(-)	(-)	(-)	(-)	26
(2*E*)-3-(3,4-dimethoxyphenyl)-1-(2-hydroxy-4-methoxyphenyl)-2-propen-1-one (11)	3.8	20.7	20	7	3-100	(-)	(-)	(-)	(-)	25
(2*E*)-3-(4-bromophenyl)-1-(2-hydroxy-4-methoxyphenyl)-2-propen-1-one (12)	5.7	12.7	23	7	3-100	(-)	(-)	(-)	(-)	32
(2*E*)-1-(4-methoxyphenyl)-3-(4-methoxyphenyl)-2-propen-1-one (13)	2.4	21.3	39	7	3-100	(-)	(-)	(-)	(-)	24
(2*E*)-3-(2,4-dimethoxyphenyl)-1-(4-methoxyphenyl)-2-propen-1-one (14)	2.7	20.8	26	7	3-100	(-)	(-)	(-)	(-)	25
(2*E*)-1-(2,4-dimethoxyphenyl)-3-(4-methoxyphenyl)-2-propen-1-one (15)	>8.6	<4.4	12	7	0.3-10	(-)	(-)	(-)	(-)	(-)
(2*E*)-3-(4-Hydroxy-3-methoxyphenyl)-2-propenoic acid 2-(3,4-dihydroxyphenyl)ethyl ester [1]	3.6	66.6	95	10	30-600	(-)	(-)	(-)	(-)	38
(2*E*)-3-(4-hydroxyphenyl)-2-propenoic acid 2-(3,4-dihydroxyphenyl)ethyl ester [2]	4.9	61.7	92	10	30-600	(-)	(-)	(-)	(-)	32
(2*E*)-3-(3,4-dihydroxyphenyl)-2-propenoic acid 2-(3,4-dihydroxyphenyl)ethyl ester [3]	14.4	23.5	50	10	30-600	(-)	(-)	(-)	(-)	(-)
(2*E*)-3-(4-hydroxy-3-methoxyphenyl)-2-propenoic acid 2-(4-hydroxyphenyl)ethyl ester [4]	1.7	104.8	78	10	30-600	(-)	(-)	(-)	(-)	38
(2*E*)-3-(4-hydroxyphenyl)-2-propenoic acid 2-(4-hydroxyphenyl)ethyl ester [5]	2.4	136	65	10	30-600	(-)	(-)	(-)	(-)	15
(2*E*)-3-(3,4-dihydroxyphenyl)-2-propenoic acid 2-(4-hydroxyphenyl)ethyl ester [6]	10.0	25.1	35	10	30-600	(-)	(-)	(-)	(-)	(-)
(2*E*)-3-phenyl-2-propenoic acid 2-(4-hydroxyphenyl)ethyl ester [7]	2.2	102.4	42	10	30-600	(-)	(-)	(-)	(-)	20
(2*E*)-3-(4-hydroxyphenyl)-2-propenoic acid 2-phenylethyl ester [8]	2.5	57.9	37	10	30-600	(-)	(-)	(-)	(-)	(-)
(2*E*)-3-(3,4-dihydroxyphenyl)-2-propenoic acid 2-phenylethyl ester [9]	23.4	8.5	25	10	3-100	(-)	(-)	(-)	(-)	26
(2*E*)-3-phenyl-2-propenoic acid 2-phenylethyl ester [10]	>2.0	196.6	120	10	30-600	(-)	(-)	(-)	(-)	18
2-phenyl-2H-1-benzopyran [1]	2.4	76.7	49	7.7	30-600	(-)	(-)	(-)	(-)	(-)
2-(4-methoxyphenyl)-2H-1-benzopyran [2]	2.8	81.9	81	7.7	30-600	(-)	(-)	(-)	(-)	12
2-(3,4-dimethoxyphenyl)-2H-1-benzopyran [3]	2.9	81.1	60	7.7	30-600	(-)	(-)	(-)	(-)	28
2-(4-fluorophenyl)-2H-1-benzopyran [4]	2.8	67.1	47	7.7	30-600	(-)	(-)	(-)	(-)	(-)
2-(4-chlorophenyl)-2H-1-benzopyran [5]	2.5	82.4	48	7.7	30-600	(-)	(-)	(-)	(-)	(-)
2-(4-bromophenyl)-2H-1-benzopyran [6]	2.2	67.8	51	7.7	30-600	(-)	(-)	(-)	(-)	9
7-methoxy-2-phenyl-2H-1-benzopyran [7]	3.1	71	82	7.7	30-600	(-)	(-)	(-)	(-)	19
7-methoxy-2-(4-methoxyphenyl)-2H-1-benzopyran [8]	4.7	73.3	61	10	30-600	(-)	(-)	(-)	(-)	30
2-(3,4-dimethoxyphenyl)-7-methoxy-2H-1-benzopyran [9]	2.9	50.6	49	10	30-600	(-)	(-)	(-)	(-)	22
2-(4-bromophenyl)-7-methoxy-2H-1-benzopyran [10]	4.0	81.1	62	10	30-600	(-)	(-)	(-)	(-)	20
(2*E*,4*E*)-5-(3,4-methylenedioxyphenyl)-2,4-pentadienoic acid (4-methoxyphenyl)methyl ester [1],	>10.5	456	51	11	30-1000	(-)	(-)	(-)	(-)	35
**(2E,4E)-5-(3,4-methylenedioxyphenyl)-2,4-pentadienoic acid (4-hydroxy-3-methoxyphenyl)methyl ester [2]**	**><1.1**	378	>1000	11	30-1000	820	640	**>1.2**	**>1.6**	**61**
(2*E*,4*E*)-5-(3,4-methylenedioxyphenyl)-2,4-pentadienoic acid 2-(4-hydroxyphenyl)ethyl ester [3]	2.6	118	61	11	30-1000	(-)	(-)	(-)	(-)	16
(2*E*,4*E*)-5-(3,4-methylenedioxyphenyl)-2,4-pentadienoic acid 2-(3,4-dihydroxyphenyl)ethyl ester [4]	8.0	137	63	11	30-1000	(-)	(-)	(-)	(-)	26
(2*E*,4*E*)-5-(3,4-methylenedioxyphenyl)-2,4-pentadienoic acid 2-(4-methoxyphenyl)ethyl ester [5]	1.6	73	38	11	10-300	(-)	(-)	(-)	(-)	(-)
(2*E*,4*E*)-5-(3,4-methylenedioxyphenyl)-2,4-pentadienoic acid 2-(3,4-dimethoxyphenyl)ethyl ester [6]	1.9	80	55	7	10-300	(-)	(-)	(-)	(-)	(-)
(2*E*,4*E*)-5-(3,4-methylenedioxyphenyl)-2,4-pentadienoic acid 2-phenylethyl ester [7]	>6.6	15	75	7	3-100	(-)	(-)	(-)	(-)	11
(2*E*,4*E*)-5-(3,4-methylenedioxyphenyl)-2,4-pentadienoic acid 3-phenylpropyl ester [8]	><1.0	75	29	7	3-100	(-)	(-)	(-)	(-)	14
(2*E*,4*E*)-5-(3,4-methylenedioxyphenyl)-2,4-pentadienoic acid 4-phenyl-2-butyl ester [9]	><1.0	>356	100	7	30-1000	(-)	(-)	(-)	(-)	13
(2*E*,4*E*)-5-(3,4-methylenedioxyphenyl)-2,4-pentadienoic acid 4-phenylbutyl ester [10]	><1.0	45	76	7	10-300	(-)	(-)	(-)	(-)	12
(2*E*,4*E*)-5-(3,4-methylenedioxyphenyl)-2,4-pentadienoic acid decyl ester [11]	><1.0	>613	105	7	30-1000	(-)	(-)	(-)	(-)	15
2-[(1*E*)-2-phenylethenyl]-4*H*-1-benzopyran-4-one [1]	2.1	37.7	42	10	3-100	(-)	(-)	(-)	(-)	18
2-[(1*E*)-2-(4-fluorophenyl)ethenyl]-4*H*-1-benzopyran-4-one [2]	8.9	19.8	140	10	3-600	(-)	(-)	(-)	(-)	25
2-[(1*E*)-2-(4-chlorophenyl)ethenyl]-4*H*-1-benzopyran-4-one [3]	20.6	10.6	100	10	3-100	(-)	(-)	(-)	(-)	16
2-[(1*E*)-2-(4-bromophenyl)ethenyl]-4*H*-1-benzopyran-4-one [4]	2.8	24.5	60	10	3-100	(-)	(-)	(-)	(-)	14
2-[(1*E*)-2-(4-methoxyphenyl)ethenyl]-4*H*-1-benzopyran-4-one [5]	**84.1**	1.9	23	23	0.3-100	(-)	(-)	(-)	(-)	28
2-[(1*E*)-2-(3,4-dimethoxy)ethenyl]-4*H*-1-benzopyran-4-one [6]	2.2	45.4	37	23	3-100	(-)	(-)	(-)	(-)	34
6-methoxy-2-[(1*E*)-2-phenylethenyl]-4*H*-1-benzopyran-4-one [7]	5.7	37.6	50	23	18-600	(-)	(-)	(-)	(-)	33
2-[(1*E*)-2-(4-fluorophenyl)ethenyl]-6-methoxy-4*H*-1-benzopyran-4-one [8]	**3.1**	67.5	86	23	18-600	ND	180	ND	**>3.3**	**54**
2-[(1*E*)-2-(4-chlorophenyl)ethenyl]-6-methoxy-4*H*-1-benzopyran-4-one [9]	21.2	14.8	85	23	3-600	(-)	(-)	(-)	(-)	(-)
2-[(1*E*)-2-(4-bromophenyl)ethenyl]-6-methoxy-4*H*-1-benzopyran-4-one [10]	5.6	15.6	32	23	3-100	(-)	(-)	(-)	(-)	19
6-methoxy-2-[(1*E*)-2-(4-methoxyphenyl)ethenyl]-4*H*-1-benzopyran-4-one [11]	**89.1**	3.8	21	23	0.3-100	(-)	(-)	(-)	(-)	(-)
2-[(1*E*)-2-(3,4-dimethoxy)ethenyl]-6-methoxy-4*H*-1-benzopyran-4-one [12]	**1.2**	256.7	>600	23	18-600	ND	370	ND	**>1.6**	**64**
7-methoxy-2-[(1*E*)-2-phenylethenyl]-4*H*-1-benzopyran-4-one [13]	2.0	115.5	68	23	18-600	(-)	(-)	(-)	(-)	(-)
2-[(1*E*)-2-(4-fluorophenyl)ethenyl]-7-methoxy-4*H*-1-benzopyran-4-one [14]	17.4	12.7	200	23	3-300	(-)	(-)	(-)	(-)	46
2-[(1*E*)-2-(4-chlorophenyl)ethenyl]-7-methoxy-4*H*-1-benzopyran-4-one [15]	0.7	68	500	8	18-600	(-)	(-)	(-)	(-)	(-)
2-[(1*E*)-2-(4-bromophenyl)ethenyl]-7-methoxy-4*H*-1-benzopyran-4-one [16]	0.4	65.8	200	8	18-600	(-)	(-)	(-)	(-)	16
7-methoxy-2-[(1*E*)-2-(4-methoxyphenyl)ethenyl]-4*H*-1-benzopyran-4-one [17]	4.0	49.5	34	8	18-600	(-)	(-)	(-)	(-)	18
-[(1*E*)-2-(3,4-dimethoxy)ethenyl]-7-methoxy-4*H*-1-benzopyran-4-one [18]	4.8	56.6	54	8	18-600	ND	350	ND	0.2	62
3-[(1*E*)-2-phenylethenyl]-4*H*-1-benzopyran-4-one [1]	2.3	145.6	160	9	30-600	(-)	(-)	(-)	(-)	(-)
3-[(1*E*)-2-(4-fluorophenyl)ethenyl]-4*H*-1-benzopyran-4-one [2]	1.1	20	47	9	3-100	(-)	(-)	(-)	(-)	27
3-[(1*E*)-2-(4-chlorophenyl)ethenyl]-4*H*-1-benzopyran-4-one [3]	0.7	5.3	12	9	3-100	(-)	(-)	(-)	(-)	(-)
3-[(1*E*)-2-(4-dimethyaminophenyl)ethenyl]-4*H*-1-benzopyran-4-one [4]	9.7	14.8	16	9	3-100	(-)	(-)	(-)	(-)	(-)
6-methoxy-3-[(1*E*)-2-phenylethenyl]-4*H*-1-benzopyran-4-one [5]	0.8	83.1	250	9	30-600	(-)	(-)	(-)	(-)	(-)
3-[(1*E*)-2-(4-dimethyaminophenyl)ethenyl]-6-methoxy-4*H*-1-benzopyran-4-one [6]	0.9	6.5	19	9	3-100	(-)	(-)	(-)	(-)	8
7-methoxy-3-[(1*E*)-2-phenylethenyl]-4*H*-1-benzopyran-4-one [7]	**301.1**	0.6	1.7	21	0.3-10	(-)	(-)	(-)	(-)	(-)
3-[(1*E*)-2-(4-fluorophenyl)ethenyl]-7-methoxy-4*H*-1-benzopyran-4-one [8]	3.7	1.1	9	21	0.3-10	(-)	(-)	(-)	(-)	27
3-[(1*E*)-2-(4-chlorophenyl)ethenyl]-7-methoxy-4*H*-1-benzopyran-4-one [9]	2.4	70.8	<30	21	30-600	(-)	(-)	(-)	(-)	13
3-[(1*E*)-2-(4-dimethyaminophenyl)ethenyl]-7-methoxy-4*H*-1-benzopyran-4-one [10]	4.6	34.2	>100	21	3-100	(-)	(-)	(-)	(-)	23
7-methoxy-3-[(1*E*)-2-(4-methoxyphenyl)ethenyl]-4*H*-1-benzopyran-4-one [11]	6.2	21.3	88	21	3-100	(-)	(-)	(-)	(-)	(-)
3-[(1*E*)-2-(3,4-dimethoxyphenyl)ethenyl]-7-methoxy-4*H*-1-benzopyran-4-one [12]	9.2	20	>10	21	0.3-10	(-)	(-)	(-)	(-)	17
7-methoxy-3-[(1*E*)-2-(3,4,5-trimethoxyphenyl)ethenyl]-4*H*-1-benzopyran-4-one [13]	3.1	30	26	10	3-100	(-)	(-)	(-)	(-)	28
3-[(1*E*)-2-(4-hydroxyphenyl)ethenyl]-7-methoxy-4*H*-1-benzopyran-4-one [14]	**182.0**	0.65	1.4	10	0.3-10	(-)	(-)	(-)	(-)	(-)
